# RETRACTION: Effect of two different techniques of arteriovenous fistula puncture on wound infection in haemodialysis patients

**DOI:** 10.1111/iwj.70115

**Published:** 2024-10-17

## Abstract

Retraction: YaoY. , MaoH. , DuZ. , and LiuJ. , “Effect of Two Different Techniques of Arteriovenous Fistula Puncture on Wound Infection in Haemodialysis Patients,” International Wound Journal 21, no. 3 (2024): e14659, 10.1111/iwj.14659.38409902 PMC10897496

The above article, published online on 26 February 2024, in Wiley Online Library (http://onlinelibrary.wiley.com/), has been retracted by agreement between the journal Editor in Chief, Professor Keith Harding; and John Wiley & Sons, Ltd. It came to the publisher's attention from a third party that a number of articles shared concerning similarities in format and structure. Following an investigation by the publisher, the retraction has been agreed on as the peer review and publishing process for this article were found to be manipulated. The authors did not respond to the notice of retraction.

Retraction: Y. Yao , H. Mao , Z. Du , and J. Liu , “Effect of Two Different Techniques of Arteriovenous Fistula Puncture on Wound Infection in Haemodialysis Patients,” International Wound Journal 21, no. 3 (2024): e14659, 10.1111/iwj.14659.38409902 PMC10897496

The above article, published online on 26 February 2024, in Wiley Online Library (http://onlinelibrary.wiley.com/), has been retracted by agreement between the journal Editor in Chief, Professor Keith Harding; and John Wiley & Sons, Ltd. It came to the publisher's attention from a third party that a number of articles shared concerning similarities in format and structure. Following an investigation by the publisher, the retraction has been agreed on as the peer review and publishing process for this article were found to be manipulated. The authors did not respond to the notice of retraction.

